# Ischemic lesions in all territories as a marker of malignant hypercoagulability

**DOI:** 10.1002/ccr3.2211

**Published:** 2019-05-22

**Authors:** Claus Z. Simonsen, Eske Roth, Thabele M Leslie‐Mazwi

**Affiliations:** ^1^ Department of Neurology Aarhus University Hospital Aarhus Denmark; ^2^ Department of Neuroradiology Aarhus University Hospital Aarhus Denmark; ^3^ Department of Neurosurgery and Neurology Massachusetts General Hospital Boston Massachusetts

**Keywords:** cancer, etiology, hypercoagulation, MRI, stroke

## Abstract

Hypercoagulability due to cancer is an overlooked cause of stroke and presents with a typical MRI pattern, that is small embolic strokes in several vascular territories.

## 1 BACKGROUND

Acute stroke imaging aims to determine the nature of the stroke (ischemic or hemorrhagic), determine the location and volume of the affected tissue, identify the presence of a vascular occlusion, and define the tissue that remains viable. The ideal imaging modality for stroke patients remains an unanswered and evolving question, with CT and MRI presenting both advantages and disadvantages. One of the prominent advantages of utilizing MRI in evaluating acute ischemic stroke is the ability to detect small strokes below the resolution of CT scanning, to determine infarct volume, and to visualize the infarct pattern. The precision of this data can inform possible etiological considerations.

We present a series of six illustrative cases who all presented with a typical, clinical stroke and had strikingly similar MRI appearances (ischemic lesions in all vascular territories) and a common stroke mechanism, namely underlying cancer, causing hypercoagulability.

## 2 MATERIALS

**Figure 1:**
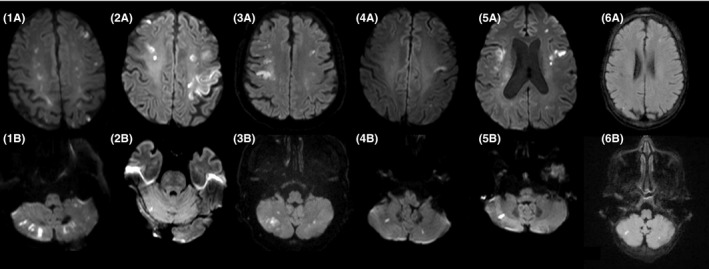
Diffusion‐weighed imaging of stroke secondary to cancer. Two images (A: supratentorial and B: infratentorial) for each of the six cases. Notice DWI lesions in cerebellum and cerebrum

The records of two large academic centers were reviewed for cases presenting with embolic stroke and malignancy. Six cases were identified (Table 1) with negative cardiac echocardiography and determination of hypercoagulability of malignancy as the cause of stroke. By Danish law, the study was exempt from notification to ethical committee and consent since the study was conducted as a part of quality improvement. IRB approval and HIPAA compliance were maintained for non‐Danish patients. A summary of the six cases is presented in Table 1. MRI scans of the patients are shown in Figure 1.

**Table 1:** Presentation of the six cases and the findings

Case	Age	Sex	Presentation	Vascular Territory	Laboratory data	ECHO	Malignancy	Direct stroke treatment	Days alive after presentation
1	79	F	Left‐sided weakness	All	Elevated basic phosphate and CRP	Normal	Adenocarcinoma, possible GI tract	None	20
2	85	F	Right‐sided weakness, aphasia	All	Elevated basic phosphate, CRP and D‐dimer		Pancreatic cancer, no biopsy	None	23
3	63	M	Left arm weakness	All		Mitral insufficiency	Lung adenocarcinoma	None	74
4	50	M	Right facial weakness	Posterior and right anterior	Elevated D‐dimer	Normal	Lung adenocarcinoma	None	44
5	77	M	Left‐sided hemiparesis	All	Elevated basic phosphate, ALAT and CRP	Normal	Pancreatic cancer	Thrombectomy	49
6	76	F	Right weakness, aphasia.	All	Elevated D‐dimer	Normal	Lung adenocarcinoma	None	28

Abbreviations: ALAT, alanine aminotransferase; CRP, C‐reactive protein; GI, gastrointestinal.

### 2.1 Case 1

A previously healthy 79‐year‐old woman presented with 14 days of nausea. She developed sudden left hemiparesis. Acute diffusion‐weighted imaging (DWI) showed patchy diffusion restriction in all cerebrovascular territories. Biochemistry revealed elevated basic phosphate 185 U/L, CRP 30 mg/L. Cerebrospinal fluid was normal. Transthoracic and transesophageal echocardiography was normal. Computed tomography (CT) of the thorax, abdomen, and pelvis showed a thrombus in the jugular vein, metastasis to mediastinal lymph nodes with compression of the superior vena cava, and metastasis to the liver and adrenal glands but no primary tumor.

Biopsy from the liver showed poorly differentiated adenocarcinoma suspected of upper gastrointestinal tract origin. The patient quickly deteriorated and died 20 days after initial presentation.

### 2.2 Case 2

A hypertensive, osteoporotic 85‐year‐old woman was found with right hemiparesis and aphasia. On MRI, she had small infarcts in all cerebrovascular territories. Biochemistry showed elevated basic phosphatase 475 U/L, with CRP 49 mL/L.

CT of the thorax, abdomen, and pelvis showed a pancreatic tumor with metastasis to the liver and adrenal glands. Surgery was not offered based on imaging findings, and chemotherapy was declined by the patient. She died 23 days after initial presentation.

### 2.3 Case 3

A 63‐year‐old man with a newly diagnosed lung cancer (adenocarcinoma) with bone and liver metastasis was scheduled for chemotherapy. He presented after the abrupt onset of left arm weakness, prior to the initiation of treatment. Acute MRI demonstrated a causative ischemic lesion and infarcts in all cerebrovascular territories. Transthoracic echocardiography demonstrated mild mitral insufficiency. He was treated with palliative chemotherapy, but died 74 days after the stroke.

### 2.4 Case 4

A 50‐year‐old male with a history of depression was a 38‐pack‐year smoker. He presented with sudden right facial paresis and dysarthria. MRI revealed small infarcts in all vascular territories. Cerebrospinal fluid was normal. Transthoracic and transesophageal echocardiography was normal.

CT of the thorax, abdomen, and pelvis showed a mass in the lung, and PET‐CT showed nodular metastasis in both lungs, metastasis to retroperitoneal lymph nodes and lymphangitis carcinomatosis. Biopsy showed adenocarcinoma.

He was started on chemotherapy but died 44 days after the initial stroke.

### 2.5 Case 5

A 77‐year‐old hypertensive male presented with sudden left‐sided hemiparesis and diffuse DWI changes in multiple vascular territories on acute MRI. MRA revealed a proximal vessel occlusion, and he underwent successful thrombectomy. Elevated basic phosphate 340 U/L and alanine aminotransferase 75 U/L, and CRP 41 mg/L were noted. Transthoracic echocardiography revealed a hypertensive heart, but was otherwise normal. CT of the thorax, abdomen, and pelvis showed a pancreatic tumor with liver metastasis, mesenteric metastasis, and a thrombus in the right pulmonary artery. Biopsy showed adenocarcinoma of the pancreas.

The patient was deemed unfit for chemotherapy and died 49 days after presentation.

### 2.6 Case 6

A 76‐year‐old female with known metastatic lung carcinoma and a history of pulmonary embolism on apixaban presented with aphasia and right‐sided weakness. Imaging with MRI revealed embolic strokes in all vascular territories. Transthoracic echocardiography was normal, with no septal defects. D‐dimer was elevated with the evidence of new thrombus on ultrasonography of the lower extremities. Family discussion leads to a conversion to hospice, and she died 28 days later.

## 3 DISCUSSION AND CONCLUSIONS

Determination of stroke etiology is a central tenant of stroke care. Embolic stroke indicates a proximal clot source that releases thrombus into the intracranial circulation, with subsequent vessel occlusion leading to cerebral tissue damage. Practically, the source can either be atherosclerosis (artery‐to‐artery emboli), the heart, or a hypercoagulable state.

Embolic stroke etiology varies widely but the pattern of embolic damage can be a clue to the source. Unilateral or single vascular territory stroke indicates that the embolic source is likely the proximal large artery (eg, the carotid or the vertebrobasilar artery). Embolic sources proximal to that can distribute emboli to all vascular territories.

Hypercoagulability secondary to malignancy is the most common paraneoplastic syndrome. This phenomenon has been long recognized; Armand Trousseau described the Trousseau's sign of malignancy, migratory thrombophlebitis because of inflammation related to thrombus formation within vessels, but now the term is extended to include disseminated intravascular coagulopathy, marantic endocarditis, and arterial emboli in patients with cancer.^1^

Certain malignancies (especially adenocarcinomas of the lung and gastrointestinal tract) are particularly associated with hypercoagulability. In stroke, this hypercoagulability can cause emboli through three predominant mechanisms: paradoxical embolization through a persistent foramen ovale in the atrium, generation of small clot aggregates on the undersurface of cardiac valves (marantic endocarditis), and hypercoagulability by secreted substances. The hypercoagulability can be conveyed by secretion of mucin (a “sticky” glycosylated molecule), tissue factor, and cancer procoagulant.^2^ The first two mechanisms would only be visible on transesophageal echocardiography. The finding of a negative transthoracic echocardiography in these cases argues for a hypercoagulable cause for stroke. Differential diagnosis with this pattern can be stroke of cardiac origin (eg, atrial fibrillation), hematological diseases, and vasculitis.^3^

Our patients did uniformly poor. They were not candidates for intravenous thrombolysis because of strokes of varying ages. One of our patients (case 5) was treated with thrombectomy since he had a sudden, witnessed, severe deficit and a large vessel occlusion, but proximal vessel occlusions were not noted in the remaining patients. While an encouraging recovery from multiple small strokes is often possible because of the large amount of viable tissue for plasticity, these patients’ outcomes were driven by the underlying malignancy.

It is well known that cancer can be a cause of deep vein thrombosis and that this condition has a poor prognosis. In one large study, a one‐year survival of only 12% was seen.^4^ Hypercoagulability is also possible on the arterial side with data suggesting as much as a risk of 7% of a cancer diagnosis after an ischemic stroke presentation.^5^ Strokes caused by cancer are known to be associated with an embolic patterns in multiple territories,^6^ more often cryptogenic^7^ and with elevated D‐dimer.^8^ Median survival in a previous study was only 96 days.^9^ D‐dimer was measured in patients 2, 4, and 6 where it was elevated in all cases.

When treating stroke due to hypercoagulability, we have often extrapolated from the studies of malignancy‐induced hypercoagulability on the venous side, where low‐molecular‐weight heparin is the drug of choice.^10^ A recent study found that a novel oral anticoagulant (edoxaban) was noninferior to low‐molecular‐weight heparin treatment.^11^ This will perhaps have implications in the arterial circulation as well.

While we have pooled data from two large tertiary centers, our study is subject to the typical limitations of a small, retrospective evaluation, prominently the absence of a denominator. We also lack the ability to directly compare findings to other forms of embolic stroke. Finally, our findings are MRI based, and the utility of MRI is limited in certain environments.

Here, we present six cases with ischemia in a strikingly similar pattern on MRI. Our goal is to raise awareness of a characteristic MRI pattern in the setting of unremarkable echocardiography findings. All patients had arterial hypercoagulability, an underlying adenocarcinoma, and all had a poor prognosis. The presence of the imaging findings described in the absence of an identified cause should raise concern for a possible primary malignancy.

- Hypercoagulability as seen in cancer can be a cause of stroke.
- Patients can present as a typical stroke patient, but MRI reveals a pattern with diffusion restriction in all vascular territories (cerebellum and cerebrum bilaterally).
- The cancer is often an adenocarcinoma with a poor prognosis.

## CONFLICT OF INTEREST

None declared.

## AUTHOR CONTRIBUTION

CZS: conceived the idea, gathered data, performed literature search, and drafted the first version of the manuscript. ER: gathered imaging and performed imaging analysis. TM L‐M: gathered data, did literature search and supervised the project. All authors edited the final manuscript.
